# Biochemical characteristics of a free cyanide and total nitrogen assimilating *Fusarium oxysporum* EKT01/02 isolate from cyanide contaminated soil

**DOI:** 10.1016/j.dib.2017.07.023

**Published:** 2017-07-17

**Authors:** Enoch A. Akinpelu, Adewole T. Adetunji, Seteno K.O. Ntwampe, Felix Nchu, Lukhanyo Mekuto

**Affiliations:** Bioresource Engineering Research Group (*BioERG*), Department of Biotechnology, Cape Peninsula University of Technology, P.O. Box 652, Cape Town 8000, South Africa

**Keywords:** Biochemistry, Cyanide, *Fusarium oxysporum*

## Abstract

Sustainability of nutrient requirements for microbial proliferation on a large scale is a challenge in bioremediation processes. This article presents data on biochemical properties of a free cyanide resistant and total nitrogen assimilating fungal isolate from the rhizosphere of *Zeamays* (maize) growing in soil contaminated with a cyanide-based pesticide. DNA extracted from this isolate were PCR amplified using universal primers; TEF1-α and ITS. The raw sequence files are available on the NCBI database. Characterisation using biochemical data was obtained using colorimetric reagents analysed with VITEK^®^ 2 software version 7.01. The data will be informative in selection of biocatalyst for environmental engineering application.

**Specifications Table**

**Table:** 

Subject area	Biology
More specific subject area	Biochemistry
Type of data	Table
How data was acquired	Biochemical assays were done using VITEK^®^ 2 Compact 30 system (BioMérieux, France)
Data format	Analysed
Experimental factors	DNA extracted from this isolate were PCR amplified using TEF1-α and ITS primers. Prior testing for free cyanide and total nitrogen assimilation was done in *Beta vulgaris* supplemented cultures [1]
Experimental features	Cyanide degrading fungi isolated from rhizosphere of *Z. mays* contaminated with cyanide-based pesticides. Colorimetric reagent cards were used for the biochemical tests.
Data source location	*BioERG* laboratory, Cape Town, South Africa (33.9324˚S, 18.6406˚E)
Data accessibility	The nucleotide sequences of the isolate is publicly available on the NCBI database with accession numbers KU985430 and KU958431

**Value of the data**

• An extended biochemical analyses of free cyanide-degrading and total nitrogen assimilating fungal isolate.
• This data presents information on the capacity of the isolate to utilise various substrates especially carbohydrates and amino acid assimilation.
• The prominent enzymes expressed by this isolate would be useful in environmental engineering applications.

## 1. Data

Data presented here contains the biochemical characteristics of *Fusarium oxysporum* EKT01/02 isolate from the rhizosphere of *Z. mays* contaminated with a cyanide-based pesticide using colorimetric reagent cards. Biochemical data from Gram negative (GN), Gram positive (GP), Gram positive spore-forming Bacilli (BCL), and Yeast and yeast-like organisms (YST) are shown in Table 1, generated using combined data from reagent cards.

**Table 1:** Biochemical reaction details.

**Test**	**Result**	**Test**	**Result**
Ala-Phe-Pro arylamidase	+	D-Amygdalin	−
Adonitol	−	Phosphatidylinositol phospholipase C	–
L-Pyrrolydonyl-arylamidase	–	D-Xylose	–
L-Arabitol	–	Arginine dihydrolase 1	–
D-Cellobiose	–	Cyclodextrin	–
β-galactosidase	–	L-Aspartate arylamidase	–
Hydrogen sulphide production	–	β-Galactopyranosidase	–
β-N-Acetyl-glucosaminidase	–	α-Mannosidase	–
Glutamyl arylamidase pNA	–	Leucine arylamidase	+
D-Glucose	–	β Glucuronidase	–
Fermentation/Glucose	–	L-Pyrrolidonyl-arylamidase	–
β-glucosidase	+	β-Glucuronidase	–
D-Maltose	–	Alanine arylamidase	+
D-Mannitol	–	D-Galactose	–
D-Mannose	–	D-Ribose	–
β-Xylosidase	–	Lactose	–
β-Alanine arylamidase	–	N-Acetyl-D-Glucosamine	–
L-Proline arylamidase	+	Growth in 6.5% NaCl	–
Lipase	–	Methyl-B-D-Glucopyranoside	–
Palatinose	–	Pullulan	–
Tyrosine arylamidase	+	D-Raffinose	–
D-Sorbitol	–	Salicin	–
Saccharose/Sucrose	–	Arginine dihydrolase 2	–
D-Tagatose	–	L-Lysine-arylamidase	+
D-Trehalose	–	Leucine-arylamidase	+
Citrate (Sodium)	–	Phenylalanine arylamidase	+
Malonate	–	L-Proline armylamidase	+
5-keto-D-Gluconate	–	Glycogen	–
L-lactate alkalinisation	–	myo-Inositol	–
Succinate alkalinisation	–	Methyl-A-D-Glucopyranoside acidification	–
β-N-Acetyl galactosaminidase	–	Methyl-D-Xyloside	–
α-galactosidase	–	Maltotriose	–
Phosphatase	–	Glycine arylamidase	+
Arginine GP	+	Acetate assimilation	+
Erythritol assimilation	+	Citrate (Sodium) assimilation	–
Glycerol assimilation	(+)	Glucuronate assimilation	+
Arbutin assimilation	–	L-Proline assimilation	–
Amygdalin assimilation	(−)	2-Keto-D-Gluconate assimilation	–
D-Galactose assimilation	(+)	N-Acetyl-Glucosamine assimilation	–
Gentiobiose assimilation	+	D-Gluconate assimilation	–
D-Glucose assimilation	+	Ornithine decarboxylase	–
Lactose assimilation	–	Lysine decarboxylase	–
Methyl-A-D-Glucopyranoside assimilation	–	L-Histidine assimilation	–
D-Cellobiose assimilation	–	Coumarate	–
ϒ-Glutamyl-transferase	+	β-Glucoronidase	–
D-Maltose assimilation	+	O/129 resistance (comp. vibrio.)	–
D-Raffinise assimilation	–	Glu-Gly-Arg-Arylamidase	–
PNP-N-acetyl-BD-galactosaminidase 1	–	L-malate assimilation	–
D-Mannose assimilation	–	Ellman	–
D-Melibiose assimilation	(+)	L-Lactate assimilation	–
D-Melezitose assimilation	–	D-Melezitose	–
L-Sorbose assimilation	+	L-Rhanose	–
L-Rhamnose assimilation	+	β-Mannosidase	–
Xylitol assimilation	–	Phosphoryl Chlorine	(+)
D-Sorbitol assimilation	–	Pyruvate	–
Saccharose/Sucrose assimilation	–	Inulin	–
Urease	+	Putrescine assimilation	–
α-Glucosidase	+	Esculin hydrolysis	+
D-Turanose assimilation	+	Tetrazolium red	–
D-Trehalose assimilation	+	Polymixin B resistance	–
Nitrate assimilation	+	Bacitracin resistance	–
L-Arabinose assimilation	+	Novobiocin resistance	–
D-Galacturonate assimilation	(−)	Optochin resistance	–
L-Glutamate assimilation	–	Kanamycin resistance	+
D-Xylose assimilation	–	Oleandomycin resistance	–
DL-Lactate assimilation	–	Polymixin_B resistance	–

## 2. Experimental design, materials and methods

### 2.1 Sample collection and identification

Fungi were isolated from soil containing cyanide-based pesticides using a culture-based technique. The fungal isolate was identified both morphologically and by structural ribosomal deoxyribonucleic acid (rDNA) sequencing analysis. The genomic DNA was extracted using a PowerBiofilm DNA kit (MOBIO Laboratories, Inc., CA- USA) according to the manufacturer's instructions. The Polymerase Chain Reaction (PCR) amplification and sequencing was done using the approach described earlier [2]. For complete identification, translation elongation factor 1-alpha (TEF1-α) and internal transcribe spacer (ITS) rDNA sequences were amplified using universal primers EF1F/EF1R (EF1F: ‘ATGGGTAAGGARGACAAGAC’ and EF1R: ‘GGARGTACCAGTSATCATGTT’) and ITS1/ITS4 (ITS1: ITS ‘TCCGTAGGTGAACCTGCGG’ and ITS4: ITS ‘TCCTCCGCTTATTGATATGC’), respectively [3]. The PCR amplicons were purified using a QIAquick PCR purification kit (Qiagen, Hilden, Germany). PCR amplicons from TEF 1-α gene were denoted EKT01 while those of ITS were denoted EKT02. Sequences were analysed using a CLC Main Workbench 7 followed by a search in the National Centre for Biotechnology Information (NCBI, www.ncbi.nlm.nih.gov) database.

### 2.2 Biochemical reaction assays

Biochemical tests were done using colorimetric reagent cards; GN (Gram negative), GP (Gram positive), BCL (Gram positive spore-forming bacilli), and YST (Yeast and yeast-like organisms) of the VITEK^®^ 2 Compact 30 system (BioMérieux, France). The inoculum was prepared from a culture of the isolate incubated at 35 °C for 18 to 24 h on Potato Dextrose Agar (Merck, USA). A sterile stick was used to transfer sufficient number of morphologically similar colonies into a 3.0 mL sterile saline (aqueous 0.4 to 0.5% NaCl) in a clear plastic test tube (12 × 75 mm). The turbidity was adjusted to a McFarland standard (0.5 to 0.63) for each card using a DensiLameter^®^. All cards were incubated on-line at 35.5 ± 1.0 °C. Periodically (15 min), each card was removed from the incubator carousel and inserted into the optical system for reaction readings at different wavelengths. The generated data were analysed using the VITEK^®^ 2 software version 7.01, according to the manufacturer's instructions. Test reaction data are shown as “+”, “−”, “(+)”, or “(-)” in Table 1. Data in parentheses indicate reactions which are weak and too close to the test threshold [4].
